# AAV1/2-induced overexpression of A53T-α-synuclein in the substantia nigra results in degeneration of the nigrostriatal system with Lewy-like pathology and motor impairment: a new mouse model for Parkinson’s disease

**DOI:** 10.1186/s40478-017-0416-x

**Published:** 2017-02-01

**Authors:** Chi Wang Ip, Laura-Christin Klaus, Akua A. Karikari, Naomi P. Visanji, Jonathan M. Brotchie, Anthony E. Lang, Jens Volkmann, James B. Koprich

**Affiliations:** 1 Department of Neurology, University Hospital of Würzburg, Josef-Schneider-Strasse 11, 97080 Würzburg, Germany; 2 Morton and Gloria Shulman Movement Disorders Centre & Edmund J. Saffra Program in Parkinson’s Disease, Toronto Western Hospital, Toronto Western Hospital, 399 Bathurst Street, 9MC 422, Toronto, ON M5T 2S8 Canada; 3 Krembil Research Institute, Toronto Western Hospital, University Health Network, 60 Leonard Avenue, 8KD402, Toronto, ON M5T 2S8 Canada; 4 Tanz Centre for Research in Neurodegenerative Diseases, University of Toronto, Toronto, ON Canada; 5 Department of Medicine, University of Toronto, Toronto, ON Canada

**Keywords:** Parkinson’s disease, α-synuclein, A53T mutation, Mouse model, Lewy-like pathology

## Abstract

α-Synuclein is a protein implicated in the etiopathogenesis of Parkinson’s disease (PD). AAV1/2-driven overexpression of human mutated A53T-α-synuclein in rat and monkey substantia nigra (SN) induces degeneration of nigral dopaminergic neurons and decreases striatal dopamine and tyrosine hydroxylase (TH). Given certain advantages of the mouse, especially it being amendable to genetic manipulation, translating the AAV1/2-A53T α-synuclein model to mice would be of significant value. AAV1/2-A53T α-synuclein or AAV1/2 empty vector (EV) at a concentration of 5.16 x 10^12^ gp/ml were unilaterally injected into the right SN of male adult C57BL/6 mice. Post-mortem examinations included immunohistochemistry to analyze nigral α-synuclein, Ser129 phosphorylated α-synuclein and TH expression, striatal dopamine transporter (DAT) levels by autoradiography and dopamine levels by high performance liquid chromatography. At 10 weeks, in AAV1/2-A53T α-synuclein mice there was a 33% reduction in TH+ dopaminergic nigral neurons (*P* < 0.001), 29% deficit in striatal DAT binding (*P* < 0.05), 38% and 33% reductions in dopamine (*P* < 0.001) and DOPAC (*P* < 0.01) levels and a 60% increase in dopamine turnover (homovanilic acid/dopamine ratio; *P* < 0.001). Immunofluorescence showed that the AAV1/2-A53T α-synuclein injected mice had widespread nigral and striatal expression of vector-delivered A53T-α-synuclein. Concurrent staining with human PD SN samples using gold standard histological methodology for Lewy pathology detection by proteinase K digestion and application of specific antibody raised against human Lewy body α-synuclein (LB509) and Ser129 phosphorylated α-synuclein (81A) revealed insoluble α-synuclein aggregates in AAV1/2-A53T α-synuclein mice resembling Lewy-like neurites and bodies. In the cylinder test, we observed significant paw use asymmetry in the AAV1/2-A53T α-synuclein group when compared to EV controls at 5 and 9 weeks post injection (*P* < 0.001; *P* < 0.05). These data show that unilateral injection of AAV1/2-A53T α-synuclein into the mouse SN leads to persistent motor deficits, neurodegeneration of the nigrostriatal dopaminergic system and development of Lewy-like pathology, thereby reflecting clinical and pathological hallmarks of human PD.

## Introduction

Parkinson’s disease (PD) is the second most common neurodegenerative disorder with pathological hallmarks consisting of degeneration of dopaminergic neurons within the substantia nigra (SN), loss of dopaminergic terminals projecting into the striatum and accumulation of insoluble protein aggregates, termed Lewy bodies and Lewy neurites. The latter two are composed of several proteins with α-synuclein (aSyn) being the most abundant component [18]. The physiologic role of aSyn remains to be completely understood with reports suggesting a function in synaptic transmitter release due to its relationship with presynaptic vesicles [1, 4]. The pathogenic role of insoluble aSyn in PD is even less clear [3], however the fact that hereditary forms of PD can be caused by duplication, triplication and missense mutations in the aSyn encoding gene (e.g. A53T [22]), A30P [16]) has strongly linked this protein to the pathogenesis of PD [10, 16, 22]. Based on these observations, numerous mouse models for PD with pathogenic aSyn expression have been generated. There are strains of transgenic mice that express human mutated A53T-aSyn, A30P-aSyn or both [7]. Although these mice mimic some aspects of the human disease, including aSyn accumulation in neurons of the SN, the major drawback in most of these models is the lack of reproducible nigrostriatal damage [7] and the timeframes to induce any degenerative changes are long and variable. In addition to transgenic PD models, several gene delivery based animal models of synucleinopathies have been generated by using lentiviral [17] and adeno-associated vectors (AAV), the latter demonstrating a high affinity for dopaminergic neurons [27, 29]. Non-human primates and rats develop PD-related histological and behavioural features from AAV driven overexpression of mutated A53T-aSyn, including early, within 6-16 weeks, dopaminergic neuron loss in the SN [9, 12–14]. In rats, injection of an AAV1/2 vector that contained human mutated A53T-aSyn cDNA provides a rapid, within 6 weeks, and progressive course of dopaminergic nigrostriatal degeneration that is accompanied by aSyn aggregates. By using a chimeric approach, this vector combines the advantages of AAV serotype 2, high neuronal tropism and ability to produce high titers, with that of AAV serotype 1, i.e. excellent ability to penetrate brain tissue [13, 14]. Attempts have been made with different AAV serotypes in mice to generate PD models but these either demonstrated resilience to aSyn-induced neurodegeneration by showing no, or only slight, dopaminergic neuronal loss after relatively long exposure times [5, 8, 25, 30] or have been so aggressive as to produce high levels of neurodegeneration without definite evidence of Lewy pathology that did not model early PD [21, 24]. In view of the advantages of knock-out or transgenic mouse models for studying molecular mechanisms of neurodegeneration and resilience our aim was to translate the AAV1/2-A53T-aSyn mediated rat PD model to the mouse system.

## Material and Methods

### Animals

43 male wild-type (wt) mice on C57BL/6 background weighting 24–30 g were purchased from Charles River Laboratories, Sulzfeld, Germany and investigated at the age of 12 weeks. Mice were kept in near pathogen-free environment under standard conditions (21 °C, 12 h/12 h light-dark cycle).

### Human midbrain sections

Human tissue was obtained from the Canadian Brain Tissue Bank, and donated for research purposes following informed consent. PD nigra was obtained from an 80-year-old male with an 11-year clinical history of PD. Post mortem analysis revealed diffuse Lewy pathology with neuronal nigral degeneration. Control tissue was obtained from a 77-year-old male with no history of neurologic disease and no evidence of neurodegenerative changes on postmortem. 4 μm sections obtained from formalin fixed paraffin embedded blocks were cut for staining.

### Adeno-associated vectors (AAV) 1/2 serotype injection

AAV1/2 were designed as described by Koprich et al., 2010 [14]. Deeply anaesthetized mice were stereotactically injected unilaterally into the right SN with a microinjector (Stoelting, Kiel, Wisconsin, USA) at a rate of 0.5 μl/min with either 1.5 μl of empty AAV1/2 (empty vector; EV) or 1.5 μl AAV1/2 expressing human mutated A53T-aSyn both at a concentration of 5.16 x 10^12^ genomic particles (gp)/ml. According to the mouse brain atlas of Paxinos and Franklin (Paxinos and Franklin, The Mouse Brain in Stereotaxic Coordinates, Second Edition, 2001) the coordinates from Bregma: AP -3.1 mm; ML -1.4 mm; DV -4.4 mm were applied for injection. 22 mice were injected with AAV1/2-A53T-aSyn, 21 mice with AAV1/2-EV.

### Behavioral studies

Spontaneous forepaw use was assessed by using the cylinder test before, 5 and 9 weeks after AAV1/2 injection. Briefly, mice were placed into a transparent plexiglass cylinder of 12 cm diameter and 30 cm height in front of two mirrors and were video recorded for a duration of 10 min. The videos were scored post-hoc by an observer blinded to the treatment condition according to the description by Schallert et al., 2000 [23]. Each rearing of the mice was analyzed for the number of touches of the inner surface of the cylinder with either the right (ipsilateral), the left (contralateral) or both forepaws simultaneously. The final data was presented as percentage of the ipsilateral (right) forepaw use by calculation with the equation: {(ipsilateral paw + 0.5 both paws) / (ipsilateral paw + contralateral paw + both paws)} x 100. The calculated percentage declares the preference of the forepaw use as follows: 50% = symmetric use of both forepaws; <50% = preference of the left forepaw; >50% preference of the right forepaw.

### Tissue processing and immunohistochemistry

10 weeks after AAV1/2 injection mice were transcardially perfused with 0.1 M phosphate buffered saline (PBS). Fresh mouse brains were dissected in coronal plane with a brain matrix slicer at the region of +0.14 mm from Bregma (figure 30 Paxinos and Franklin mouse brain atlas). The ventral part including the ventral striatum was snap frozen in liquid dry ice-cooled isopentane for HPLC analysis, dopamine transporter (DAT) autoradiography and immunostaining for dopaminergic terminals. The dorsal part including the middle and dorsal striatum as well as the SN was immersion-fixed in 4% paraformaldehyde (PFA) in 0.1 M PBS for two days and cryo-protected in 30% sucrose/0.1 M PBS solution for another 2 days followed by freezing of the tissue in liquid dry ice-cooled isopentane.

Tyrosine hydroxylase (TH) staining was used to label dopaminergic fibers in 10 μm coronal cryo-sections of the striatum (Paxinos and Franklin: figure 23). Sections were preincubated for 30 min in 5% normal bovine serum (BSA) in 0.1 M PBS and then incubated with rabbit anti-mouse TH antibody (abcam, Cambridge, UK) diluted in 1% BSA/PBS for overnight at 4 °C. To visualize the primary antibody, a biotinylated secondary antibody to rabbit Igs was applied for 1 h, followed by avidin/biotin reagent (Dako, High Wycombe, UK) before incubation and staining with diaminobenzidine-HCl (DAB) and H_2_O_2_.

For unbiased stereology 40 μm PFA-fixed cryo-sections were serially cut in the coronal plane. Two series were preincubated for 1 h in 10% normal goat serum (NGS)/2% BSA/0.5% Triton X-100 in 0.1 M PBS on a shaker and then incubated with either rabbit anti-mouse TH (abcam, Cambridge, UK) or mouse anti-NeuN antibody (Merck, Millipore, CA, USA) diluted in 2% NGS/2% BSA/0.5% Triton X-100 in 0.1 M PBS for overnight at 4 °C. Secondary antibodies against either rabbit or mouse Igs was applied for 2 h followed by avidin/biotin reagent and incubation and staining with DAB and H_2_O_2_.

For TH/aSyn immunofluorescence double staining, PFA-fixed sections were blocked with 10% NGS and 2% BSA for 1 h at room temperature (RT). Sections were then simultaneously incubated overnight at 4 °C with rabbit anti-mouse TH (abcam, Cambridge, UK) and mouse anti-human aSyn (Invitrogen, Maryland, USA). This was followed by incubation with fluorescence labelled goat anti-mouse Cy2 and goat anti-rabbit Cy3 secondary antibodies (Jackson ImmunoResearch Laboratories Inc. Pennsylvania, USA) for 2 h at RT. DAPI nuclear staining (Sigma Missouri, USA) was subsequently performed at RT for 20 min.

### Analysis of dopaminergic fibers and unbiased stereology

Density of TH+ fibers was measured in striatum by optical density analysis (NIH ImageJ software) after transforming colour into 8-bit greyscale images and calculated as the difference between right and left striatal optical density and the non-specifically stained corpus callosum. Four striata slices per mouse were analyzed.

The optical fractionator method of the Stereo Investigator software package (version 11.07; MicroBrightField Biosciences, Williston, VT) was used for unbiased stereological estimation of dopaminergic and total neuronal numbers (NeuN+) in the SN. The investigator was blinded to treatment groups. Seven sections per animal on average extending the rostral to caudal portions of the SN pars compacta and reticulata were used for quantification. The sections were separated by 160 μm (1/4 series) and TH-immunoreactive neurons in both SN pars compacta and reticulata were included within each selected region. Counting parameters were: grid size 110 x 110 μm, counting frame 50 x 50 μm and 2 μm guard zone. Actual mounted thickness was determined by randomly selecting sections and determining thickness at three counting sites. Sections were analyzed with a 100x/1.25 numerical aperture objective on a BX53 microscope (Olympus). Gundersen coefficient of error for m = 1 were all less than or equal to 0.09 for each section counted.

### Autoradiography

Striatal DAT level were examined from fresh-frozen tissue sections using [^125^I]-RTI-121 binding autoradiography as described [13]. In brief, slides first were incubated in binding buffer containing 50 mM Tris, 120 mM NaCl and 5 mM KCl at RT. Afterwards the sections were placed in the same buffer with additional 50 pM [^125^I]-RTI-121 (Perkin-Elmer, specific activity 2200 Ci/mmol) for a duration of 2 h at 25 °C. We defined non-specific binding as that observed in the presence of 100 mM GBR 12909 (Tocris Bioscience). Slides were washed in ice-cold binding buffer, followed by rinsing in ice-cold distilled water and dried. After this slides were apposed to autoradiographic film (Kodak) together with [125I]- microscale standards (Amersham) and left for 2d at 4 °C before development. Analysis of autoradiograms was done by using MCID software (Image Research Inc, Ontario, Canada). Densitometric examination of three striata slices per mouse was conducted with a reference curve of c.p.m. versus optical density was calculated from b- emitting [14C] micro-scale standards and used to quantify the intensity of signal as nCi/g. Moreover, the background intensity was subtracted from each reading. In the end, data was expressed as mean ± SEM. signal intensity for the treatment groups. In 0.1% a non-specific binding was found.

### Catecholamine quantification by high performance liquid chromatography (HPLC)

Analysis was performed by sending tissue pieces to CMN/KC Neurochemistry Core Lab in Vanderbilt University for HPLC analysis. Brain sections were homogenized in 200–750 μl of 0.1 M TCA (10^−2^ M sodium acetate, 10^−4^ M EDTA, 10.5% methanol). Samples were centrifuged at 10000 g for 20 min. Supernatant was removed, the pellet saved for protein analysis. Catecholamines were analyzed by using a specific HPLC assay with an Antec Decade II (oxidation: 0.5) electrochemical detector operated at 33 °C. Supernatant samples were injected by utilizing a Water 717+ autosampler onto a Phenomenex Nucleosil (5u, 100A) C18 HPLC column (150x4.60 mm). Then, analytes were eluted with a mobile phase (89.5% 0.1 M TCA, 10^−2^ M sodium acetate, 10^−4^ M EDTA, 10.5% methanol) followed by delivery of the solvent at 0.8 ml/min with a Waters 515 HPLC pump. Analytes were examined in the following order: 3,4-dihydroxyphenylacetic acid (DOPAC), dopamine (DA), homovanillic acid (HVA). For HPLC control and data acquisition Waters Empower software was used. Total protein for each sample was determined (Peirce BCA protein assay). Catecholamines values were expressed as ng analyte/mg total protein.

### Immunohistochemistry in human and mouse SN and striatum reveals Lewy-like pathology

4 μm formalin-fixed paraffin-embedded human brain tissue sections were dewaxed in 5 changes of xylene then along with 40 μm formalin-fixed mouse brain sections were brought down to water through graded alcohols. Sections were exposed to proteinase K (Enzo Life Sciences, Farmingdale, NY) (1:100 for 15 min at 37 °C water followed by 5 min at room temperature). Antigen retrieval was performed using citrate buffer (98 °C for 20 min followed by 20 min at room temperature). Endogenous peroxidase activity was quenched with 3% hydrogen peroxide.

To detect human aSyn, sections were incubated with primary antibody LB509 (Thermo Fisher Scientific, cat# 180215) at a dilution of 1:500 overnight at 4 °C. For detection of the phosphorylated form of aSyn at serine 129 (pSer129) sections were incubated in formic acid for 5 min before proteinase K digestion as described above, prior to incubation with primary antibody 81A (Biolegend, cat #825701) at a dilution of 1:8000 for 1 h at room temperature. Following incubation with secondary antibodies staining was detected using an ImmPRESS Alkaline Phosphatase polymer kit (Vector Laboratories, Burlington, ON) and colour development with Warp Red (Biocare Medical, Concord, CA). Finally, sections were counterstained with Mayer’s Hematoxylin, dehydrated in alcohols, cleared in xylene and mounted.

### Exclusion of animals from analysis

Seven animals of the AAV1/2-A53T-aSyn group were excluded from analysis after verification of an unsuccessful AAV1/2 injection into the SN by evaluation of double immunofluorescence stainings against TH/aSyn depicted by lack of co-localization of the A53T aSyn protein with TH+ dopaminergic neurons in the SN. For technical reasons one AAV1/2-EV exposed mouse was excluded from the DAT autoradiography analysis; one AAV1/2-A53T-aSyn exposed mouse was excluded from analysis of DA metabolites (2 mice for AAV1/2-A53T-aSyn DOPAC) another mouse from AAV1/2-EV group (2 mice DOPAC).

### Statistical analysis

For statistical analysis the distribution of each set of values was investigated via Q-Q-plots. For normal distributed data parametric methods were utilized, in case of different variances the Welch’s correction was used, for non-normal distributed data non-parametric methods were employed as statistical tests. For the cylinder test, analysis of striatal TH+ optical density, stereological estimation of SN cell counts the parametric two-tailed *t*-test was used, the analysis of DAT binding was performed with Welch’s *t*-test because of different variances. In case of HPLC measurements non-parametric Mann-Whitney test was implemented. *P < 0.05, **P < 0.01, ***P < 0.001 were considered as significant P-values.

## Results

### Unilateral AAV1/2-A53T-aSyn delivery into the substantia nigra leads to behavioral deficits in mice

**Fig. 1:**
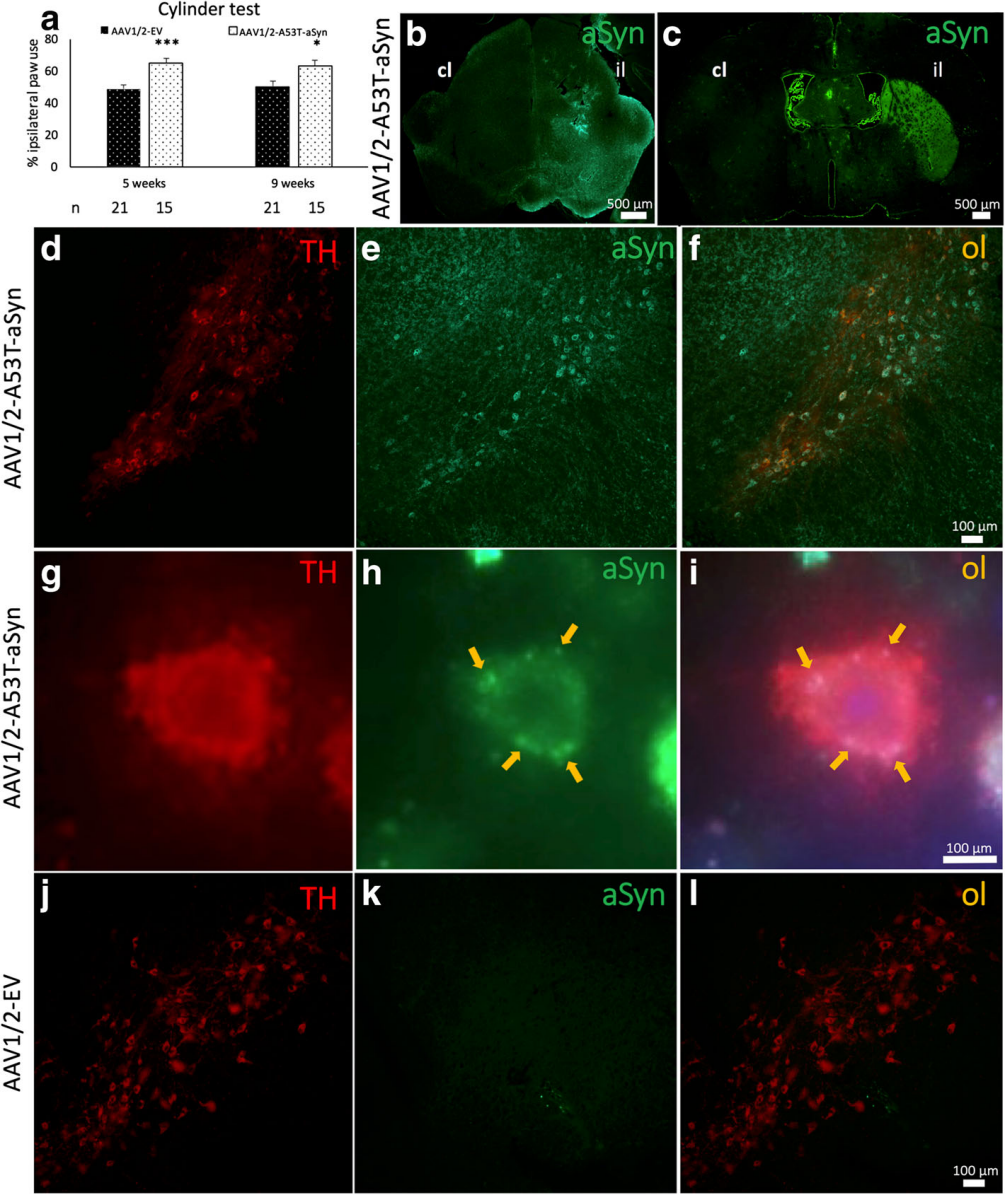
AAV1/2-A53T-aSyn SN injection leads to motor deficits and widespread aSyn expression and aggregation. **a** Unilateral AAV1/2-A53T-aSyn delivery leads to a significant asymmetry of forepaw use with preference of the ipsilateral forepaw relative to the side of injection as compared to AAV1/2-EV control mice. This behavioral deficit was visible at 5 and 9 weeks after AAV1/2 injection (mean ± SEM). **b** By immunofluorescence staining of mouse midbrain sections, widespread human A53T-aSyn protein (*green*) could be detected in the ipsilateral SN (il) 10 weeks after AAV1/2-A53T-aSyn injection (cl = contralateral). **c** Mutated A53T-aSyn protein (*green*) was found in the ipsilateral striatum (il) 10 weeks after delivery of AAV1/2-A53T-aSyn in the SN thus indicating a transport of this protein along the nigrostriatal tract. **d**-**f** Double immunofluorescence staining of AAV1/2-A53T-aSyn treated mouse SN depicting TH+ dopaminergic neurons (*red*) (**d**) and A53T-aSyn transgenic protein (*green*) (**e**) that co-localize (**f**). **g**-**i** High magnification of a TH+ dopaminergic neuron in the SN (*red*) (**g**). The same neuron is stained for mutated A53T-aSyn (*green*) (**h**). Aggregates of mutated A53T-aSyn protein can be detected in the cytoplasm of this neuron (arrows) (**h**, **i**). **j**-**l**) TH+ neurons that were injected with AAV1/2-EV (**j**) do not express human A53T-aSyn after 10 weeks (**k**, **l**). *P < 0.05; ***P < 0.001

To examine if unilateral AAV1/2-A53T-aSyn injection results in a measurable behavioural deficit in mice, we assessed motor behavior by the cylinder test at two time points, 5 and 9 weeks, during the 10 week in-life phase of the experiment. A significant asymmetry of forepaw use with preference for the right side (ipsilateral to AAV1/2-A53T-aSyn injection) was found in AAV1/2-A53T-aSyn mice compared to the AAV1/2-EV control group (mean ± SEM; week 5: AAV1/2-A53T-aSyn 65 ± 3% and AAV1/2-EV 48 ± 2%, t(34) = 4.313, *P* = 0.0001; week 9: AAV1/2-A53T-aSyn 63 ± 4% and AAV1/2-EV 50 ± 3%, t(34) = 2.335, *P* = 0.0256) (Fig. 1a).

### Degeneration of nigrostriatal pathways after AAV1/2-A53T-aSyn injection into the substantia nigra

AAV1/2-A53T-aSyn delivery into the SN resulted in a widespread expression of the transgene covering the whole SN (Fig. 1b). Ten weeks after AAV1/2-A53T-aSyn injection, a corresponding A53T-aSyn labeling of ipsilateral striatal fibers was detected (Fig. 1c). At the same time most of the remaining TH+ SN neurons co-localized with human A53T-aSyn as depicted by double immunofluorescence staining thus showing a production of the A53T-aSyn transgene by dopaminergic neurons (Fig. 1d-f). Moreover, human aSyn + aggregates were located in the cytoplasm of TH+ SN cells (Fig. 1g-i). As expected, human A53T-aSyn expression was not observed in mice after EV delivery (Fig. 1j-l).

**Fig. 2:**
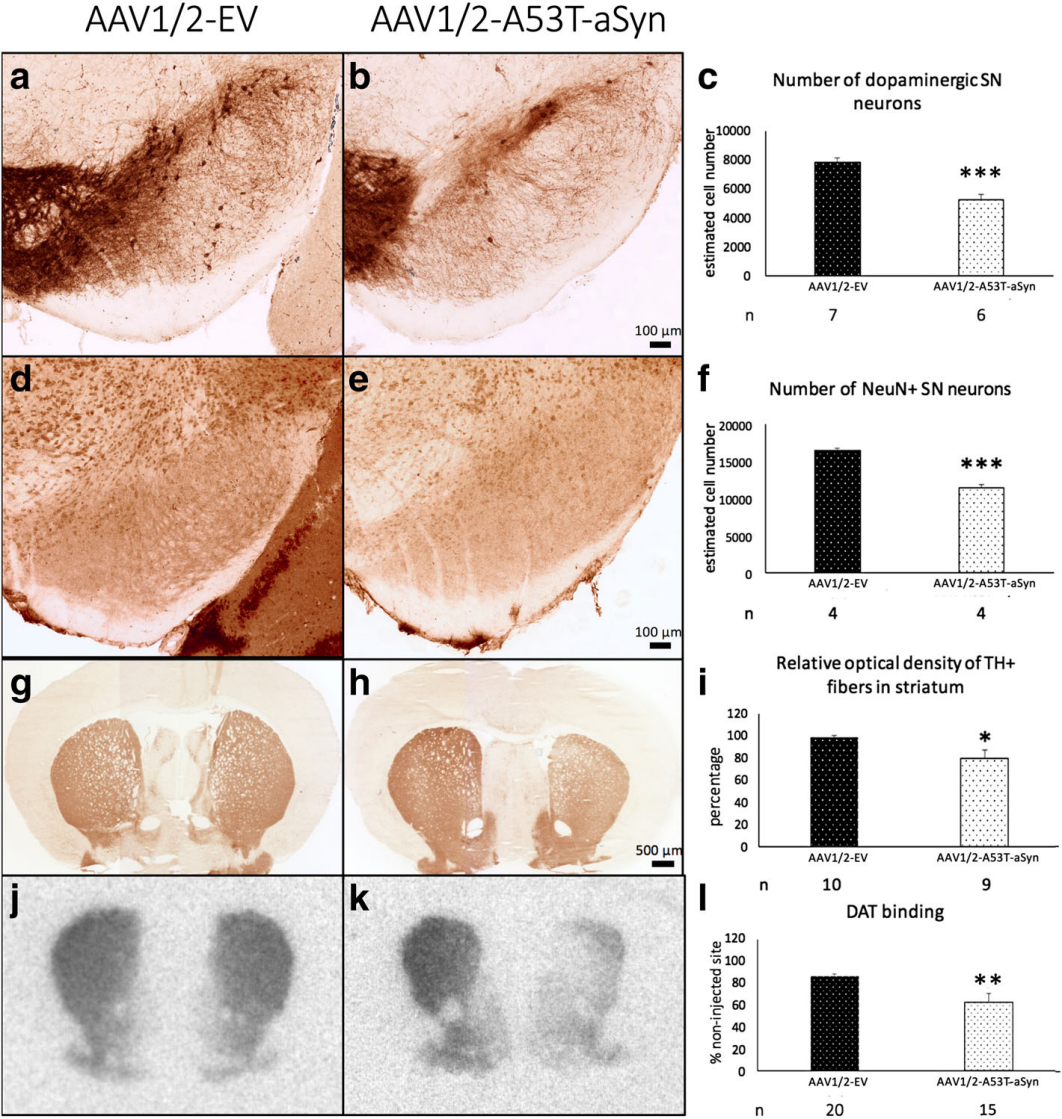
AAV1/2-A53T-aSyn administration in the SN causes neurodegeneration in the nigrostriatal tract. **a**-**c** Immunohistochemical staining of the injected, right SN for TH+ dopaminergic neurons. 10 weeks after administration of AAV1/2 into the SN a significant difference of TH+ dopaminergic neuronal cell number is visible comparing AAV1/2-EV (**a**) to AAV1/2-A53T-aSyn treated mice (**b**). AAV1/2-A53T-aSyn (white bar) induces a significant reduction of TH+ neurons by ~33% as compared to AAV1/2-EV mice (black bar) (mean ± SEM) (**c**). **d**-**f**) At the same timepoint a significant reduction of total NeuN+ neurons by ~31% was found in AAV1/2-A53T-aSyn injected mice as compared to AAV1/2-EV control mice (mean ± SEM). **g**-**i** TH immunohistochemical staining of AAV1/2-EV delivered striatum (**g**). AAV1/2-A53T-aSyn injection in the SN leads to an ipsilateral decrease of the optical density by ~20% as compared to AAV1/2-EV mice (mean ± SEM) (**h**, **i**). **j**-**l**) Images of striatal DAT binding by autoradiography reflects comparable reduction of striatal dopaminergic fibers by ~29% in AAV1/2-A53T-aSyn as compared to AAV1/2-EV treated mice (mean ± SEM). *P < 0.05; **P < 0.01; ***P < 0.001

The extent of nigrostriatal degeneration after AAV1/2-A53T-aSyn injection was determined (10 weeks post AAV1/2 injection) by quantification of SN neuronal cells (dopaminergic, by TH labeling and total neuron population by NeuN labeling) and dopaminergic innervation of striatum (TH immunoreactivity and DAT binding) (Fig. 2). Unbiased stereology revealed that the estimated number of TH+ dopaminergic neurons in the ipsilateral SN was significantly lower in AAV1/2-A53T-aSyn treated mice compared to AAV1/2-EV injected controls: 5270 ± 374 cells and 7903 ± 271 cells, respectively (mean ± SEM; t(11) = 5.810, *P* = 0.0001) (Fig. 2a-c). Quantification of total neuronal number in the SN showed a corresponding, significant reduction of neurons in the ipsilateral SN of AAV1/2-A53T-aSyn mice (11434 ± 441 and AAV1/2-EV 16461 ± 640; t(6) = 6.460; *P* = 0.0007) (Fig. 2d-f). Additionally, we investigated the relative optical density of TH+ striatal dopaminergic fibers (Fig. 2g-i). In AAV1/2-EV exposed mice the optical density of TH+ fibers of the ipsilateral, injected side was 98 ± 5% relative to the contralateral side, while in contrast, AAV1/2-A53T-aSyn injection led to a decrease of the ipsilateral striatal TH+ optical density to 79 ± 7% relative to the contralateral side. Comparing these results, a significant reduction of the TH+ optical density in AAV1/2-A53T-aSyn mice by 20% from AAV1/2-EV mice was evident (t(17) = 2.269, *P* = 0.0366). Additionally, analyses of DAT binding by autoradiography revealed a significant reduction of presynaptic DAT in AAV1/2-A53T-aSyn injected striatum by 29% from AAV1/2-EV treated mice (mean ± SEM; AAV1/2-A53T-aSyn 62 ± 8% and AAV1/2-EV 87 ± 2%, t(16.09) = 3.063, *P* = 0.0074) (Fig. 2j-l).

### Reduction of striatal neurotransmitter level and increase of dopaminergic metabolite turnover in AAV1/2-A53T-aSyn injected mice

**Fig. 3:**
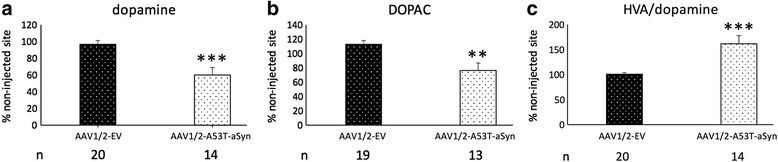
AAV1/2-A53T-aSyn SN delivery results in reduction of DA and higher DA turnover. **a**-**c** Diagrams show HPLC analyses of DA and dopaminergic metabolite levels of mouse striatum. Each bar represents the relative level in percent comparing the right (injected) side to the contralateral (non-injected side). 10 weeks after AAV1/2 injection a significant reduction of DA level is visible in AAV1/2-A53T-aSyn mice (*white* bar) as compared to the AAV1/2-EV group (black bar) (**a**). A significant reduction of DOPAC is also found in AAV1/2-A53T-aSyn treated animal (**b**). The elevated ratio of HVA/DA (**c**) indicates a higher DA turnover in AAV1/2-A53T-aSyn mice. **P < 0.01; ***P < 0.001

For examination of dopaminergic neurotransmitter and metabolite levels, striatal HPLC was performed and analyzed for DA and the metabolites DOPAC and HVA (Fig. 3). Ten weeks after AAV1/2-A53T-aSyn SN injection a significant reduction of striatal DA level to 60 ± 9% relative to the contralateral side was observed. This represented a significant decrease of 38% compared to the EV control group, 96 ± 4% of the contralateral side (*P* < 0.001) (Fig. 3a). Additionally, a decrease of DOPAC of 33% was seen in AAV1/2-A53T-aSyn treated mice (112 ± 6%) as compared to EV controls (76 ± 10%; *P* < 0.01) (Fig. 3b). At the same time point elevated HVA/DA level of 60% (162 ± 16% vs 101 ± 3%) was found in AAV1/2-A53T-aSyn expressing mice indicative for a significantly higher DA turnover compared to the AAV1/2-EV group (*P* < 0.001) (Fig. 3c).

### AAV1/2-A53T-aSyn mice develop Lewy-like pathology

**Fig. 4:**
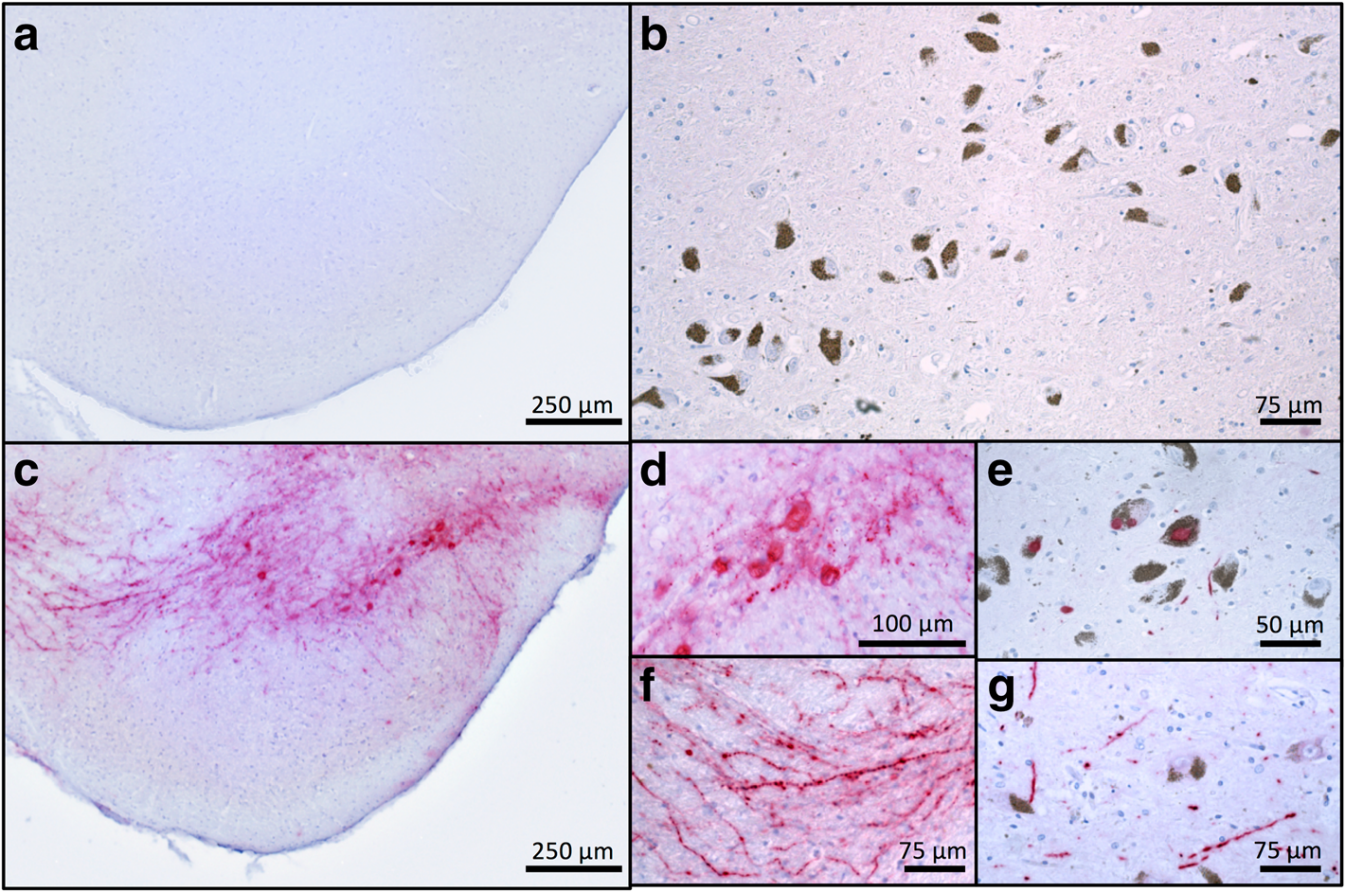
AAV1/2-A53T-aSyn SN develop insoluble aSyn + aggregates with Lewy-like pathology. **a**-**g** Immunhistochemical staining for aSyn with Lewy body specific LB509 antibody after proteinase K digestion. While AAV1/2-EV control (**a**) and human control SN (**b**) do not show any positive aSyn labelling, AAV1/2-A53T-aSyn injected mice maintain a widespread aSyn positivity after proteinase K digestion (**c**) that is located in perikarya (**d**) and in neurites (**f**). From a morphological perspective, these findings resemble the situation found in human PD sections corresponding to a Lewy-like pathology (**e**, **g**)

**Fig. 5:**
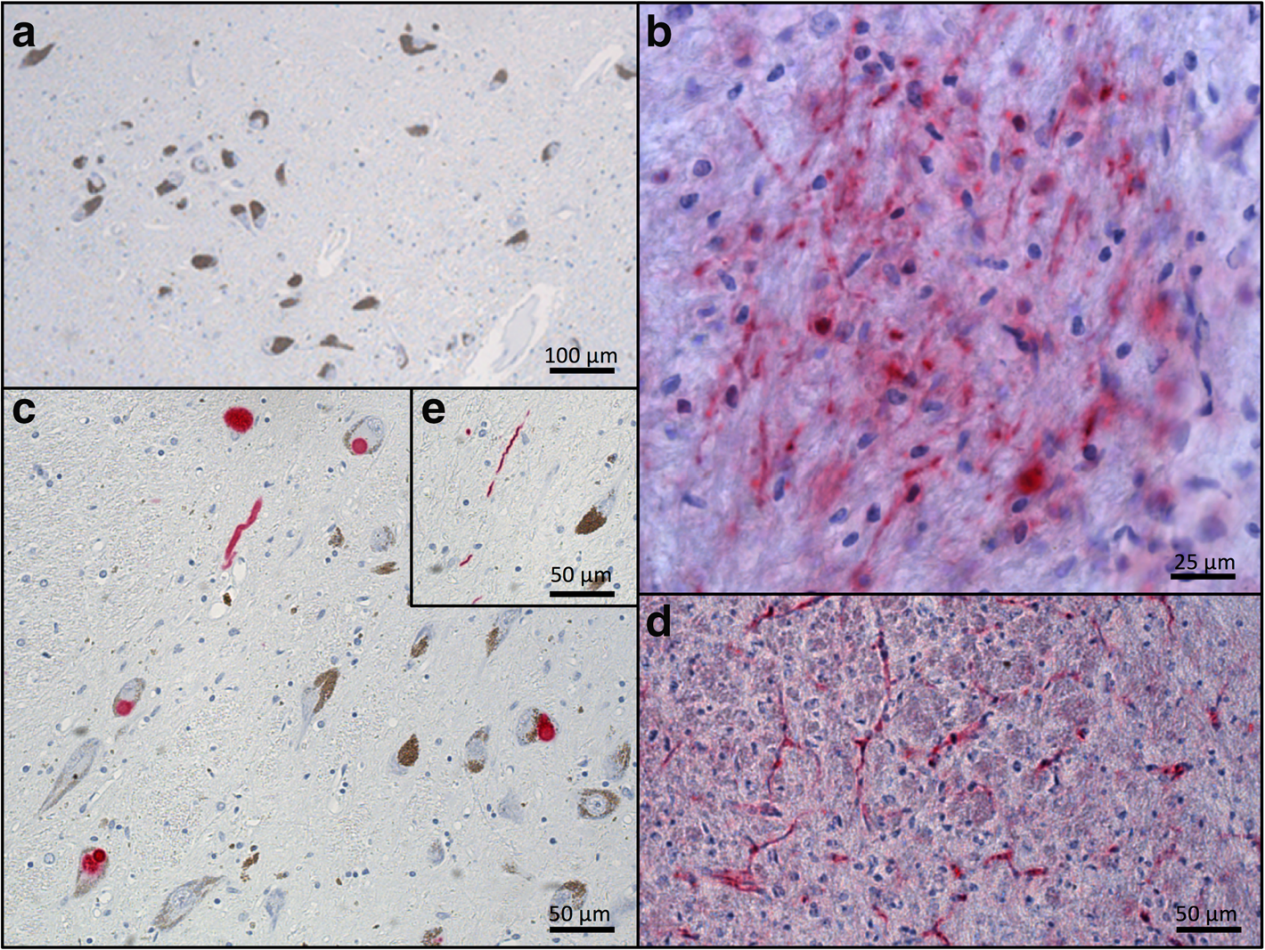
AAV1/2-A53T-aSyn SN develop insoluble pSer129 aSyn with Lewy-like pathology. There was no positive immunostaining for pSer129 aSyn in human control nigra (**a**). In contrast, in animals injected with AAV1/2-A53T-aSyn there was a strong signal for pSer129 aSyn in the SN of the ipsilateral hemisphere including several densely labelled cells (**b**). Human PD nigral Lewy bodies stained with the same method are provided for comparison (**c**). Similarly, several structures were noted in mouse brain (**d**) with morphology consistent with Lewy neurites in human PD brain (**e**)

**Fig. 6:**
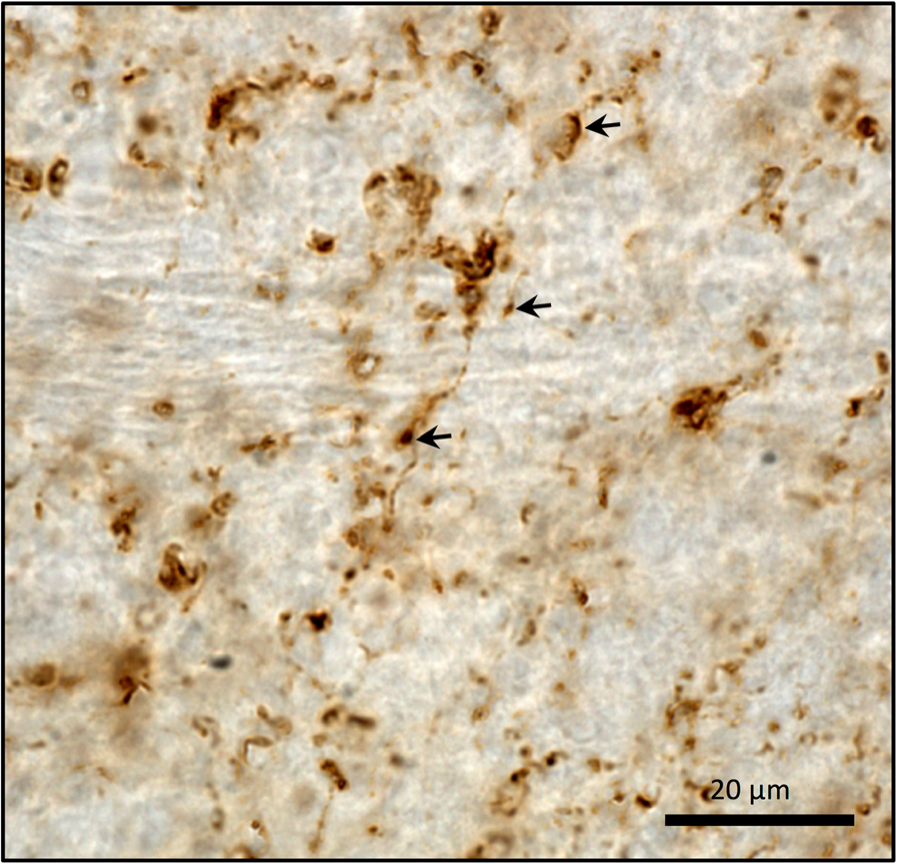
AAV1/2-A53T-aSyn striatum demonstrates LB509+ dystrophic neurites. Striatum of AAV1/2-A53T-aSyn injected mice were stained with antibody directed against LB509. LB509+ aSyn dystrophic neurites (arrows) are shown

There was no positive immunostaining by the LB509, raised against human Lewy body aSyn, in mouse brain injected with AAV1/2-EV (Fig. 4a) or in human control SN (Fig. 4b) indicating that the immunohistochemical methods used to demonstrate human aSyn (including proteinase K digestion) do not detect physiological aSyn. In contrast, in animals injected with AAV1/2-A53T-aSyn there was a strong signal for aSyn in the SN of the ipsilateral hemisphere (Fig. 4c). High power microscopy revealed several densely labelled cells within the mouse SN (Fig. 4d). Human PD nigral Lewy bodies stained using the same method are provided for comparison (Fig. 4e). Similarly, several structures were noted in mouse brain (Fig. 4f) exhibiting a morphology consistent with Lewy neurites in human PD brain (Fig. 4g). Additionally, staining for Ser129 phosphorylated aSyn after proteinase K digestion was performed (Fig. 5). In human control SN no positive immunostaining for pSer129 aSyn was detected, indicating that the immunohistochemical method used does not detect any endogenous physiologic phosphorylated Ser129 aSyn (Fig. 5a). In contrast, AAV1/2-A53T-aSyn injected mouse SN showed densely labelled pSer129 aSyn positive cells (Fig. 5b) resembling pSer129 aSyn positive cells in human PD brain (Fig. 5c). Moreover, pSer129 aSyn positive Lewy-like neurites were observed in AAV1/2-A53T-aSyn mouse SN (Fig. 5d) as well as in human PD tissue (Fig. 5e). Furthermore, aSyn (LB509) positive dystrophic neurites were detected in the striatum of AAV1/2-A53T-aSyn injected mice (Fig. 6)

## Discussion

The aim of this study was to generate a mouse model for PD that develops behavioural deficits and histopathological hallmarks resembling the human disease within a practical experimental time frame. We demonstrated that unilateral injection of AAV1/2 containing human mutated A53T-aSyn into the SN induced a widespread overexpression of A53T-aSyn in dopaminergic SN neurons that led to neurodegeneration, dopaminergic fiber loss and decrease of dopaminergic neurotransmitter in the striatum within 10 weeks. Moreover, the degeneration of the nigrostriatal system was concomitant with early motor impairment in the cylinder test at 5 weeks after AAV1/2 injection. This was accompanied by accumulation of human aSyn aggregates in the SN. The novelty of this model though is the presence of structures that are similar to Lewy bodies and neurites from human PD brain, from a morphological perspective.

Our findings that AAV1/2 can induce A53T-aSyn overexpression in dopaminergic SN neurons are comparable to previous data from the PD rat model generated by SN injection of the same AAV1/2 viral vector. Moreover, as with the related rat PD model, transport of the A53T-aSyn from the SN neurons to the striatum was observed in AAV1/2-A53T-aSyn injected mice, indicating that the A53T-aSyn protein has the capability to be transported along the nigrostriatal tract [14]. Thus, as in the AAV1/2-A53T-aSyn rat model, we could show that the AAV1/2 construct was highly effective in mice regarding the coverage of the SN DA neurons and its axonal transport along the nigrostratal tract.

There is high construct validity in the AAV-aSyn models of PD. They produce degeneration and dysfunction based on the core molecular feature of the clinical disease: toxicity associated with accumulation of aberrant aSyn. The definitive detection of pathological aSyn or Lewy bodies/neurites in the SN and other structures (e.g. amygdala, locus coeruleus) involves applying a gold standard histological methodology [2] utilizing proteinase K digestion [20] and LB509 antibody labeling procedures. In human, a positive result in this assay provides support for the postmortem diagnosis of PD. We applied this method of pathological aSyn detection in midbrain tissue sections derived from the mouse model alongside a confirmed human PD case and an aged matched control. We show that the aged-matched control case did not show any detectable levels of aSyn, likely due to clearing of physiological aSyn by proteinase K digestion. In contrast, the PD case showed abundant aSyn positive inclusions within melanized SN neurons and the presence of aSyn positive neurites. Similarly, the mouse tissue showed extensive aSyn accumulation in structures resembling both SN neurons and neurites. Thus, we demonstrate that in the AAV1/2-A53T-aSyn mouse, the SN develops proteinase K resistant structures with a morphology resembling Lewy pathology observed in humans, highlighting the histopathological similarity of aggregates induced in this mouse PD model to those in human PD. Although cytoplasmic aSyn aggregates of cell bodies have been observed in other PD mouse models, histological insolubility of these aggregates and explicit morphological similarity to human PD Lewy pathology was not shown [21, 24]. Phosphorylation of aSyn at Ser129 is considered a specific marker of α-synucleinopathies that promotes aSyn fibril formation [11]. Additionally, it has been shown that misfolding and hyperphosphorylation of aSyn might lead to central locomotor dysfunction in the (Thy1)- h[A30P]αSyn transgenic mouse model for an α-synucleinopathy [19]. We therefore analyzed the AAV1/2-A53T-aSyn mouse for Ser129 aSyn phosphorylation and found positive profiles in the SN again demonstrating the authenticity of pathologic aSyn aggregates in this mouse PD model.

We also found a significant degeneration of dopaminergic SN neurons, with a 33% reduction in AAV1/2-A53T-aSyn treated mice as compared to the EV control group. This was accompanied by significant loss of nigrostriatal dopaminergic fibers measured by striatal TH staining and DAT binding, reduced by 20% and 29%, respectively, thus recapitulating a histopathological hallmark of human PD and providing support for the model showing a high degree of face validity. Interestingly, in human PD, the onset of nigrostriatal degeneration precedes the occurrence of motor impairment with an estimated ~30% loss of SN neurons, and an even higher loss of striatal terminals, evident prior to motor symptom onset [15] (for review see Cheng et al., 2010 [6]). In contrast, in rodent models of PD, motor features can develop in the absence of significant overt nigrostriatal degeneration [13], perhaps due to the relatively intense burden of synucleinopathy in the otherwise intact nigrostriatal dopaminergic system. Indeed, although several AAV overexpression-based PD mouse models exist, either with overexpression of mutated or non-mutated aSyn, one of the main problems most of these models face so far is that they express, at best, limited nigrostriatal degeneration. Thus, two AAV models overexpressing either mutated A53T-aSyn (serotype not mentioned) [8] or human wt-aSyn (AAV2) [28] did not show degeneration of SN dopaminergic neurons even after 7 and 12 weeks post AAV injection. Similarly, in St. Martin et al. (2006), expression of human wt-aSyn by AAV (serotype not mentioned), produced no significant dopaminergic SN neuron loss after 12 weeks nor striatal dopaminergic fiber denervation of the striatum after 24 weeks [25]. In contrast, Yasuda et al. (2009), however, were able to show that AAV1-produced human wt-aSyn, delivered to the mouse SN, resulted in a significant loss in SN DA neurons at 8 weeks, however, no deficits were shown at the level of the striatum [30]. These reports are opposed to the finding in our PD model with a decrease of 38% in DA and 33% in DOPAC levels in the AAV1/2-A53T-aSyn delivered hemisphere compared to control EV injected mice. Two AAV aSyn based PD mouse models, with an AAV2 component, have, in a manner similar to the model we present here, presented a clear loss of dopaminergic SN neurons in a relatively short time frame after AAV administration. In the first study, Oliveras-Salva et al. (2013) showed, in a viral concentration dependent manner, that AAV2/7 (wt and A53T-aSyn) delivery resulted in loss of TH+ SN cells that was observed as early as 4 weeks (by 57%) with a maximal loss at 8 weeks after injection (by 82%). This was accompanied by a reduction in TH+ immunoreactivity in the striatum [21]. In the second study, Song et al. (2015) showed that AAV2/1-human wt-aSyn exposed mice showed a significant reduction in SN DA neurons after 8 weeks (by 34%) and 12 weeks (by 50%) that was accompanied by a decrease of striatal TH+ fibers and DA levels in the affected hemisphere, compared to AAV2/1-GFP controls [24]. Interestingly, and in contrast to our own report here, in the AAV2/7 and AAV2/1 models, a significant deficit in behavior was only found at later stages, from 12 weeks on, i.e. after the degeneration had occurred, and indeed, at a level exceeding what would be expected in PD patients upon first observation of motor symptoms. This is reminiscent of the 6-hydroxydopamine and 1-Methyl-4-phenyl-1,2,3,6-tetrahydropyridin toxin based models which require such levels of degeneration in order to produce spontaneous motor impairments.

There are limitations to this study. Firstly, the model necessitates a level of expertise to be able to deliver AAV1/2, by intracerebral injection, to the mouse SN, thus in our hands we mistargeted the SN in 16.3% of the cases. Secondly, reversibility of behavioural deficits using clinically defined treatments (e.g. L-DOPA, ropinirole) was not conducted and thereby the models predictive validity has yet to be determined. Thirdly, this study does not include a control group expressing a control protein. It has already been shown in the AAV1/2-A53T-aSyn rat PD model that a high titer of AAV1/2 containing green fluorescent protein (AAV1/2-GFP) causes neurodegeneration but significantly less than compared to AAV1/2-A53T-aSyn treated rats. Moreover, no loss of striatal TH-immunoreactivity was observed in AAV1/2-GFP rats, thus indicating that the toxicity of AAV1/2-GFP was not responsible for all the A53T-aSyn induced damage [14]. Nonetheless, it cannot be excluded that in the AAV1/2-A53T-aSyn mouse model the demise of dopaminergic neurons is at least to some extent independent from pathologic A53T-aSyn. Finally, although LB509 positive dystrophic neurites have been detected in the striatum of AAV1/2-A53T-aSyn mice, pathological, insoluble aSyn deposition in presynapses, that have been seen in A53T-aSyn transgenic mice [26], have not been addressed in this work.

## Conclusions

In summary, with misfolded aSyn as a key player in the pathogenesis of PD, the need for a mouse PD model with a Lewy-like α-synucleinopathy is clear. We show that the AAV1/2-A53T-aSyn mouse fulfills several criteria required of a viral vector mediated PD model, that is a) sustained production of the transgene in dopaminergic neurons, b) transport of AAV1/2 produced pathological aSyn to the striatum, c) degeneration of the nigrostriatal tract, d) histopathological similarities to human PD (Lewy-like pathology), e) behavioral deficits and f) relatively short timeframe to produce behavioral deficits and postmortem endpoints (within 2 months). This novel PD model is the first, to our knowledge, reporting all these characteristics, and is now in a position to be further analyzed and transferred to transgenic and knockout mice for unravelling molecular mechanisms of PD and preclinical testing of disease modifying therapies.
